# Outcomes in failed Primary Peripartum Hysterectomy for massive postpartum heamorrhage as compared to patients undergoing Peripartum Hysterectomy with internal iliac artery ligation

**DOI:** 10.12669/pjms.41.7.8174

**Published:** 2025-07

**Authors:** Farnaz Zahoor, Saida Abrar, Syed Muhammad Hamid

**Affiliations:** 1 Farnaz Zahoor, FCPS, CHPE, CHR, Department of OBGYN, MTI Lady Reading Hospital, Peshawar KPK, Pakistan; 2 Saida Abrar, FCPS Fellowship Urogynaecology and Pelvic Reconstructive Surgery, CHR, Department of OBGYN, MTI Lady Reading Hospital, Peshawar KPK, Pakistan; 3 Syed Muhammad Hamid, MPhil Statistics and GCP Certified Khyber Medical College, Peshawar, Pakistan

**Keywords:** Internal iliac artery ligation, Massive postpartum haemorrhage, Peripartum hysterectomy

## Abstract

**Objective:**

To evaluate the outcomes in patients undergoing peripartum hysterectomy alone for massive primary postpartum haemorrhage (Peri-hyst) compared to a patient undergoing Emergency peripartum hysterectomy with bilateral internal iliac artery ligation. (Peri-hyst + BIIAL)

**Methods:**

It was a cross-sectional comparative study conducted over two years from April 2021 to March 2023, conducted in OBGYN department of Lady Reading Hospital, Peshawar, KPK. The primary outcome was the failure of primary procedure in both group to control blood loss and required relaparotomy. Group-1 were patients undergoing pripartum hysterectomy alone and was compared with Group-2 of the patient in whom bilateral internal iliac artery ligation was also carried out at the time of Peripartum hysterectomy (Peri-hyst +BIAAL). Secondary outcomes analyzed were complications like damage to structure like ureters or sigmoid colon, internal iliac vein injury and haematoma formation in both procedures.

**Results:**

When we compare the mean outcomes of both groups by t test, it is seen that group-I had more ICU admission 15(57.7%) verses 11(42.3%), more maternal morbidity in ICU and more maternal deaths Five (55.6%) versus 4(44.4%) massive heamorrhage 25(65.8%)versus 13(34.2%), acute renal failure, acute respiratory failure p=0.818.

**Conclusion:**

Failed primary surgical management to control haemorrhage without BIIAL leads to grave outcomes as seen in most admission in ICU and maternal deaths. Early resort to IIAL is vital for improving the patient outcome when they present with massive haemorrhage.

## INTRODUCTION

Peripartum haemorrhage remains one of the major causes of maternal death in both developed and developing countries and the third-highest direct cause of maternal death (6.6 deaths/million maternity) according to the UK Seventh Report of the Confidential Enquiries into Maternal Deaths (2003–2005). Besides mortality, it is also a significant cause of maternal morbidity as seen in almost all ’near miss’ audits in both developed and developing countries.^1^-^4^

In Scotland, the rate of life-threatening haemorrhage, defined as “blood loss of 2.5 liters or more or women who receive more than five units of blood transfusion or women who receive treatment for coagulopathy after an acute event” is estimated to be 3.7/1000 maternities.^5^ The essential treatment for major PPH is medical management. When first-line treatment fails, surgical therapies should be used to control bleeding and avoid maternal death. The use and timing of second-line invasive therapies are less standardized and vary widely. Thus recommendations on treatment strategies are based largely on observational data and consensus.

Surgical methods to control massive postpartum haemorrhage include hysterectomy and pelvic devascularisation of complicated internal iliac artery ligature. Hysterectomy is the ultimate measure for controlling bleeding and preventing maternal death five while Bilateral internal iliac artery ligation procedures require a highly skilled obstetrician or vascular surgeon. Studies have shown various factors lead to PPH ending in hysterectomy, the most common being uterine rupture, uterine atony and morbidly adherent placenta%.^6^,^7^ The technique of internal iliac (hypogastric) arterial ligation to control pelvic haemorrhage is more than a century old. Howard Kelly first pioneered ligation of the internal iliac (hypogastric) artery in 1893 in the treatment of inoperative bleeding from cervical cancer later in 1963 this procedure was greatly investigated by Burchell RC and now American College of Obstetricians and Gynaecologists continued to advocate the use of bilateral internal iliac artery ligation in the management of inoperative intractable haemorrhage during pelvic surgery or cases of obstetric haemorrhage.^4^

During a massive pelvic haemorrhage or peripartum bleeding, bilateral ligation of the IIA reduces the pelvic arterial blood flow by 49% and pulse pressure by 85%.^8^ After bilateral ligation of IIA in the long-term period, the collateral circulation will maintain the re-functioning of the IIA. The deep femoral artery is the principal vascular supply to provide re-vasculature to the IIA. Anastomosis between the medial femoral circumflex and obturator artery, and the lateral femoral circumflex and superior gluteal artery are the main connection areas.^9^ Additionally, the ovarian artery also provides blood flow to the uterus. Despite bilateral ligation of the IIA, future reproductive potential is not affected totally and term pregnancies have also been reported in the literature.^10^

IIA ligation has not gained widespread popularity, primarily due to limited surgical training and concerns regarding possible complications, including buttock claudication, impotence, and urinary bladder and rectum necroses. The rationale of this study is that in our low-resource country where maternal deaths are highest due to massive haemorrhage and four delays, so carrying out peripartum hysterectomy alone cannot stop haemorrhage in already DIC patients so additional training of residents in this procedure of BIIAL should be an integral part of obstetrics and gynaecology curricula as it can reduce morbidity and mortality.

**Fig.1:**
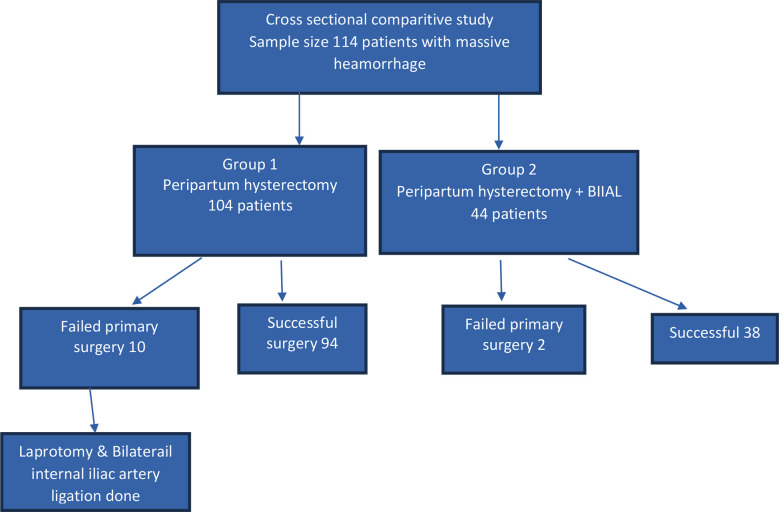
Flowchart of methodology of study.

## METHODS

It was a cross-sectional comparative study of two years from April 2021 to March 2023, conducted in the OBGYN department of Lady Reading Hospital, Peshawar, KPK. Patients enrolled in this study had undergone abdominal hysterectomy with or without internal iliac artery ligation for obstetrical indications like intractable postpartum haemorrhage secondary to ruptured uterus, placenta Previa, placenta accreta spectrum, covelier, atonic uterus and DIC. Approval from hospital Ethical Board reference number 43-1/LRH/MTI dated: February 11, 2020 was taken. In Group-1 all peripartum hysterectomy patients were included while in Group-2 hysterectomy along with bilateral internal iliac artery ligation were included. Informed consent of the patient to be enrolled in the study was taken once the patients were stable. Data was taken regarding age, parity, gestation age, mode of delivery, cause of PPH, a surgical procedure performed, outcome of primary surgery, preoperative and postoperative Hb to assess the amount of blood loss, intraoperative blood loss, number of blood products transfused, period of surgery, duration of surgery performed, duration of stay in hospital, and ICU, number of blood products transfused, status at time of discharge and complication.

The primary outcome was the failure of the primary procedure in terms of the cessation of blood loss. Secondary outcomes were admission to ICU, length of stay in the hospital, blood loss, status at the time of discharge and intraoperative complications like damage to structures like ureters, sigmoid colon and internal iliac vein.

## RESULTS

Postpartum haemorrhage (PPH) is about 3.58% of total obstetrical cases in the department and about 18.1% failed to respond to medical management and were treated by 2^nd^ line invasive surgical procedure i.e. hysterectomy. A total of 144 patients who underwent a peripartum hysterectomy were enrolled in the study. When all patients were analyzed the mean age was 30.78 ± 5.35. Demographic details are mentioned in Table-I. In Group-1, 71.2% had a primary successful surgery while 10 (83.3%) had failed surgery and underwent repeat laparotomy for internal iliac ligation. The top two causes of PPH seen were Placenta Accreted Spectrum and ruptured uterus each 38 (26.4%). Out of 144 peripartum hysterectomies, 135 (93.8%) patients survived and discharged. A total of nine (6.3%) patients died after the procedure. The number of ICU admissions was 26 (18.1%) out of which 17 (11.8%) were due to respiratory failure, four (2.8%) were due to renal failure and multiple organ failure (Table-IIA).

**Table-I:** Overview of Variables (N=144).

Name of Variable	
***Causes of PPH***	
Atonic Uterus	59 (41%)
Placentsa Previa	9 (6.3%)
Placenta Acreta Spectrum	38 (26.4%)
Rupture	38 (26.4%)
***Types of Surgery Performed***	
TAH	104 (72.2%)
TAH Plus BIIAL	40 (27.8%)
***Outcoume of Primary Surgery***	
Successful	132 (91.7%)
Failed	12 (8.3%)
***Mode of Delivery***	
Vaginal	61 (42.4%)
Caesarean Section	83 (57.6%)
***Postoperative Complications***	
Illiac Vain Rupture	1 (0.7%)
Blood Loss Anaemia	15 (10.4%)
Trauma of Bladder	11 (7.6%)
Acute Renal Failure	6 (4.2&)
Cardiac Arrest	4 (2.8%)
Respiratory Failure	8 (5.6%)
Wound Infection	2 (1.4%)
None	97 (67.4%)
***Status of time of Discharge***	
Alive	135 (93.8%)
Dead	9 (6.3%)
***ICU Admission***	
Yes	26 (18.1%)
No	118 (81.9%)
***Cause of Admission***	
Respiratory Failure	17 (11.8%)
Cardiomyopahty Undiagnosed	1 (0.7%)
Renal Failure	4 (2.8%)
MOF	4 (2.8%)
Pulmonary Embolism	1 (0.7%)
Blood Loss	1 (0.7%)
None	116 (80.6%)

**Table-IIA:** Chi-Square and Fisher test.

Variables	Subgroup	Types of surgery performed		Statistics	
		TAH	TAH PLUS BIIAL	χ² (DF)	P-value
		N (%)	N (%)		
Outcome of primary surgery	Successful	94(71.2)	38(28.8)	0.81(1)	0.369
	Failed	10(83.3)	2(16.7)		
Mode of delivery	NVD	43(70.5)	18(29.5)	0.16(1)	0.691
	C/Section	61(73.5)	22(26.5)		
Status at time of discharge	Survived	99(73.3)	36(26.7)	1.33(1)	0.249
	Dead	5(55.6)	4(44.4)		
ICU admission	Yes	15(57.7)	11(42.3)	3.34(1)	0.068
	No	89(75.4)	29(24.6)		
Admission for surgery	Elective	27(71.1)	11(28.9)	0.04(1)	0.851
	Emergency	77(72.6)	29(27.4)		
Blood Loss	Normal<1000	23(71.9)	9(28.1)	1.23(2)	0.542
	PPH 1000-2500	56(75.7)	18(24.3)		
	Masive PPH >2500	25(65.8)	13(34.2)		

Comparing the mean outcomes of both groups by t-test, it is seen that Group-1 had more ICU admission 15 (57.7%) versus 11 (42.3%), more maternal morbidity in ICU and more maternal deaths five (55.6%) versus four (44.4%). However, the amount of blood loss during surgery was less as compared to Group-2 by 188.2ml i.e. 2004.3 ± 952.8ml verses 2192.5 ± 1087 ml and the time of primary surgery was prolonged in Group-2 due to the additional procedure of BIIAL by 14.5minutes i.e. 110.6± 32.2 min verses 96.1 ± 27.4 min p-value 0.008. Blood products were also transfused more in Group-2, 5 ± 3.1 versus 4 ± 2.1 with p value 0.02 (Table-IIB).

**Table-IIB:** Comparison of variables between two groups t-test.

	Types of surgery performed		Statistics	
	TAH N=104	TAH PLUS BIIAL N=40	t-test DF=142	P-value
	Mean ± SD	Mean ± SD		
Age	30.6 ± 5.5	31.3 ± 5.1	-.66	.513
Gestation age	36.8 ± 2.4	36.2 ± 2.5	1.30	.195
Duration of stay in hospital	5.8 ± 3.2	6.2 ± 3.8	-.74	.461
Blood loss in ml	2004.3 ± 952.8	2192.5 ± 1087.8	-1.02	.310
Blood Product	4 ± 2.1	5 ± 3.1	-2.29	.024
Surgery time	96.1 ± 27.4	110.6 ± 32.2	-2.69	.008

In Group-1, about 10 patients (83.3%) had failed primary hysterectomy surgery and had reopening laparotomy in which BIIAL was done. In this group, 75% had severe blood loss anemia with massive transfusion, a complication of trauma to the bladder 50%, and ended up in ICU with acute renal injury and respiratory failure.

## DISCUSSION

The incidence of PPH in this study was 3.58%. The worldwide frequency of PPH in different populations varies from 1.2% to 12.5%, with highest frequency. The major cause of PPH in this study was uterine atony 59 (41%) patients followed by uterine rupture 38 (26.4%), this result is consistent with 80% cases of PPH due to uterine atony in frequency seen in low-income countries.^11^,^12^ The total number of emergency peripartum hysterectomies in this study reached 144, an incidence rate of 3.5/ 10 000 (95% confidence interval) births which is almost same to another study.^13^ and 0.44/1000 deliveries.^14^ In lower middle-income countries, uterine rupture (44.5%, 95% CI 36.6-52.7) was the most common indication for hysterectomy; placental pathology (48.4%, 95% CI 43.5-53.4) was most frequent in high-income settings.^15^,^16^ In this study both uterine rupture and placenta accreta spectrum had same incidence i.e. 38(26.4%). Morbidly adherent placenta has replaced uterine atony as the leading indication for emergent hysterectomy in some institution.^17^ But in this institution it has equalized the incidence of uterine rupture and it may even surpassed it due to leading trend seen over time. In study by Sumati et al., peripartum hysterectomies done were mostly due to PAS i.e. 66%.^18^

ICU admission rate for peripartum hysterectomy was 18.1% in this study with severe morbidity of 20.8% while in study by Maraschini et al.^19^ Intensive care unit admission was reported in 49.9% of cases, 16.8% of women suffered severe morbidity and five women died. When subgroup analysis is done, Group-1 versus Group-2, had ICU admission rate of 10.4% versus 7.6% with mortality rate in ICU admitted patient was 3.5% versus 2.8% respectively. Therefore, IIAL had reduced the morbidity and mortality .

In a study by Bulbul et al.^20^ the surgical time of peripartum hysterectomy was 134.2±32.3min which is almost equal to current study, the length of hospitalization was 6.1±4.6 day was also equal to this study of 6.2±3.8 days, the need for re-laprotomy was 14% while in current study was 57.7% in Group-I. In Group-I versus Group-2 patients, the surgical time (96.1 ± 27.4 min versus 110.6 ± 32.2 min p<0.008), blood products transfusion (4±2.1 versus 5±3.1 p<0.024), blood loss in ml (2004.3±952.8 versus 2192.5±1087.8, p<0.310) and duration in hospital in days (5.8 ±3.2 versus 6.2 ±3.8) were shorter. Reason may be time taken by Obstetrician consultant to perform additional surgery of internal iliac artery ligation, which usually takes standard time of 34 (26-41) min as seen in study by Grzegorz R et al.^21^

The mean blood loss during the operation and the length of hospitalization after the operation, with or without internal iliac artery ligation (IIAL) were not significantly different in study by Iwata et al.^22^ IIAL facilitates hysterectomy and prevents reactionary haemorrhage. Bleeding arrested by IIAL did not recur to require later laparotomy in any woman as seen in study.^23^,^24^ In current study the failed primary surgery was seen in Group-I in 10 patients (83.3%) versus Group-2, 2 (16.7%) 95% CI p<0.115. These patients were reoperated and BIIAL done in Group-1 and In Group-2, there were two patients had re-laparotomy in which pelvic compression packing was done as there was no active bleeder seen and patients were in DIC. Those patients with failed primary surgery, suffered morbitity and more ICU admission. Iwata et al. showed that the efficacy of IIAL was 96.87% with no intraoperative or ischemic complications. In this study the efficacy was 97.7% with just one case of internal iliac vein damage which was managed intraoperatively and patient survived. The greater the time interval between onset of hemorrhage and IIAL, the graver the outcome seen. This is seen in another study. Early resort to IIAL is vital for improving the patient outcome. When group-1 failed cases were reviewed there was much higher morbididty with 75% versus 25% more blood loss anemia, massive heamorrhage in 25 (65.8%) versus 13 (34.2%), acute renal failure in two versus 0, acute respiratory failure p=0.8.

### Strength of study:

The results of the study can guide obstetricians in optimizing the timing, technique, and patient selection for hysterectomy in massive PPH cases, potentially reducing complications. Thus institutional protocol can be developed for improving patient outcomes.

### Limitations:

It is a single center data which is conducted in LRH, so the results may not be representative of different tertiary hospital in KPK. Differences in surgical techniques and expertise among surgeons performing hysterectomies could impact morbidity outcomes, introducing a confounding factor.

## CONCLUSION

Failed primary surgical management to control haemorrhage without BIIAL leads to grave outcomes as seen in most admission in ICU and maternal deaths. Early resort to IIAL is vital for improving the patient outcome when they present with massive haemorrhage.

### Authors Contribution:

**FZ:** Conceived, designed and did editing of manuscript, is responsible for integrity of research. **SMH:** Literature search, Did statistical analysis.

**SA:** Critical review and final approval of manuscript.
